# Spatial Dynamics of the Fermi Level in Electrolyte-Gated Graphene

**DOI:** 10.1021/jacs.5c17855

**Published:** 2026-02-17

**Authors:** Iryna Ivanko, Martin Jindra, Otakar Frank, Matěj Velický

**Affiliations:** † 86875 J. Heyrovský Institute of Physical Chemistry, Czech Academy of Sciences, Dolejškova 2155/3, 182 23 Prague 8, Czech Republic; ‡ Department of Physical Chemistry, University of Chemistry and Technology in Prague, Technická 3, 166 28 Prague 6, Czech Republic

## Abstract

Understanding how electric fields propagate in nanomaterials is essential for optimizing their performance in electronic, energy, and sensing devices that require precise control of charge carrier density. We use *in situ* Raman spectroscopy combined with local voltage application via an electrolyte microdroplet to investigate the Fermi level dynamics in monolayer graphene. We observe a sharp initial shift of the Fermi level toward the charge-neutral Dirac point when crossing the biased microdroplet interface to the adjacent unbiased graphene, followed by a gradual equilibration extending tens of micrometers. Notably, the Fermi level does not fully recover to its undoped state within this range. We attribute these long-range, remote gating effects to the intrinsically low density of states of graphene, which limits its ability to screen the electric field, allowing the potential to equilibrate gradually beyond the biased region. This work introduces a robust and broadly applicable experimental platform with practical implications for semiconducting and semimetallic electronic devices.

## Graphical abstract

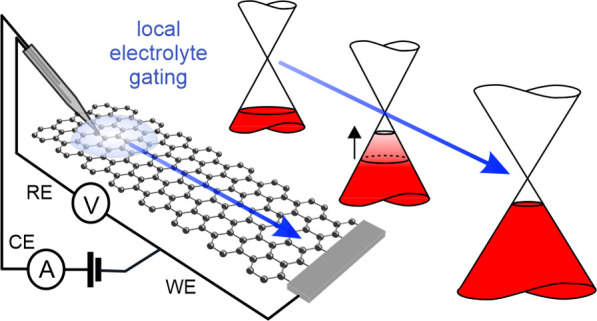

The strong coupling of graphene to external fields and the resulting modulation of its electronic band structure make it suitable for field-effect transistors,^1^ chemical or biological sensors,^2,3^ and optoelectronic devices.^4^ The tunability of its Fermi level (*E*_F_), which directly reflects the charge carrier density, shapes not only graphene’s electrical transport properties^5^ but also its optical conductivity.^6,7^

Raman spectroscopy is a rapid, nondestructive technique used to monitor charge doping^8–11^ and electron–phonon/electron–electron interactions^10,12,13^ in graphene. Intentional charge-doping methods utilize electrostatic gating with solid-state dielectrics and liquid electrolytes.^14^ Crucially, the nanometer-thick electrical double layer formed at the graphene-electrolyte interface results in capacitance of tens of μF/cm^2^, enabling modulation of carrier density using low voltages.^8,9,15–19^ Unintentional doping of graphene, caused by interactions with the substrate, adsorbates such as water or oxygen,^20^ or contaminants introduced during fabrication,^21^ also shifts *E*_F_ and obscures the intrinsic material properties.

To fully exploit charge-doping effects, one must understand how the electric field propagates through graphene under local gating. Theoretical calculations showed that intercalating species generate nonuniform charge carrier distribution in the graphite host over short length scales, strongly affecting its electronic properties.^22–24^ Scanning near-field optical microscopy revealed that local electrostatic gating affects graphene regions micrometers away from the biased region, possibly due to the in-plane electric field and the polarization of adsorbed water molecules.^25^

In order to investigate the impact of localized gating on the spatial dynamics of the charge carriers *in situ*, we combine a charge injection via an electrolyte microdroplet with Raman spectroscopy, which measures the spatial distribution of the *E*_F_ in graphene. We use mechanically exfoliated monolayer graphene, whose structural integrity enables reliable detection of spatial variations in *E*_F_. The absence of the Raman D band at ≈1350 cm^–1^, the spatial homogeneity of the Raman G and 2D band frequencies (ω_G_ and ω_2D_), and the ω_2D_–ω_G_ correlation collectively confirm the pristine, defect-free lattice of the as-prepared samples (Supporting Figure S1). The average strain and charge doping vary across different samples and fall within the ranges of –0.1% to +0.3% and –7 × 10^12^ cm^–2^ to –2 × 10^13^ cm^–2^, respectively. Raman spectroscopy is used to monitor charge carrier dynamics independently of electrolyte gating.

The voltage is applied locally through the microdroplet while the surrounding graphene remains unbiased, enabling us to track how *E*_F_ evolves outside the gated region. We observe a sharp initial shift at the droplet boundary, followed by a gradual, distance-dependent recovery. This is consistent with graphene being driven out of equilibrium inside the droplet and relaxing toward the original, substrate-induced charged state outside the droplet. Notably, this equilibration does not restore the original undoped state, reflecting graphene’s limited density of states, which supports a residual electric field that persists tens of micrometers beyond the biased region.

The Raman spectra of graphene underneath the microdroplet show uniform doping for the applied potentials from –200 to +1200 mV (Figure 1a–b). With increasing potential (hole doping), ω_G_ upshifts from the charge neutrality point (CNP) due to the removal of the Kohn anomaly from the Brillouin zone center.^12,26,27^ The CNP, equivalent to the Dirac point of graphene where the carrier density is zero, occurs at ≈−200 mV, where the ω_G_ reaches a minimum of 1584 cm^–1^ and full width at half-maximum (FWHM) of the G band (Γ_G_) reaches a maximum of 15 cm^–1^. The average CNP minimum of ω_G_ is 1581.5 ± 3.6 cm^–1^ for all measured samples, which agrees with the literature.^10,28,29^ The negative CNP potential indicates that graphene is p-doped, as expected from the literature.^30^ The ω_G_ shift from its minimum at the Dirac point (*Δω*_G_ = ω_G_ – ω_G,Dirac_) reaches ≈8 cm^–1^ at +500 mV, accompanied by the narrowing of Γ_G_ (Figure 1b). Above +600 mV, the G band splits into two components, attributed to intact (G_I_) and defective (G_D_) graphene (Supporting Figure S2), the latter of which is formed by (photo)­electrochemical oxidation.^31^ This splitting also increases the apparent G band line width. The D band is absent except for the two highest potentials.

**Figure 1:**
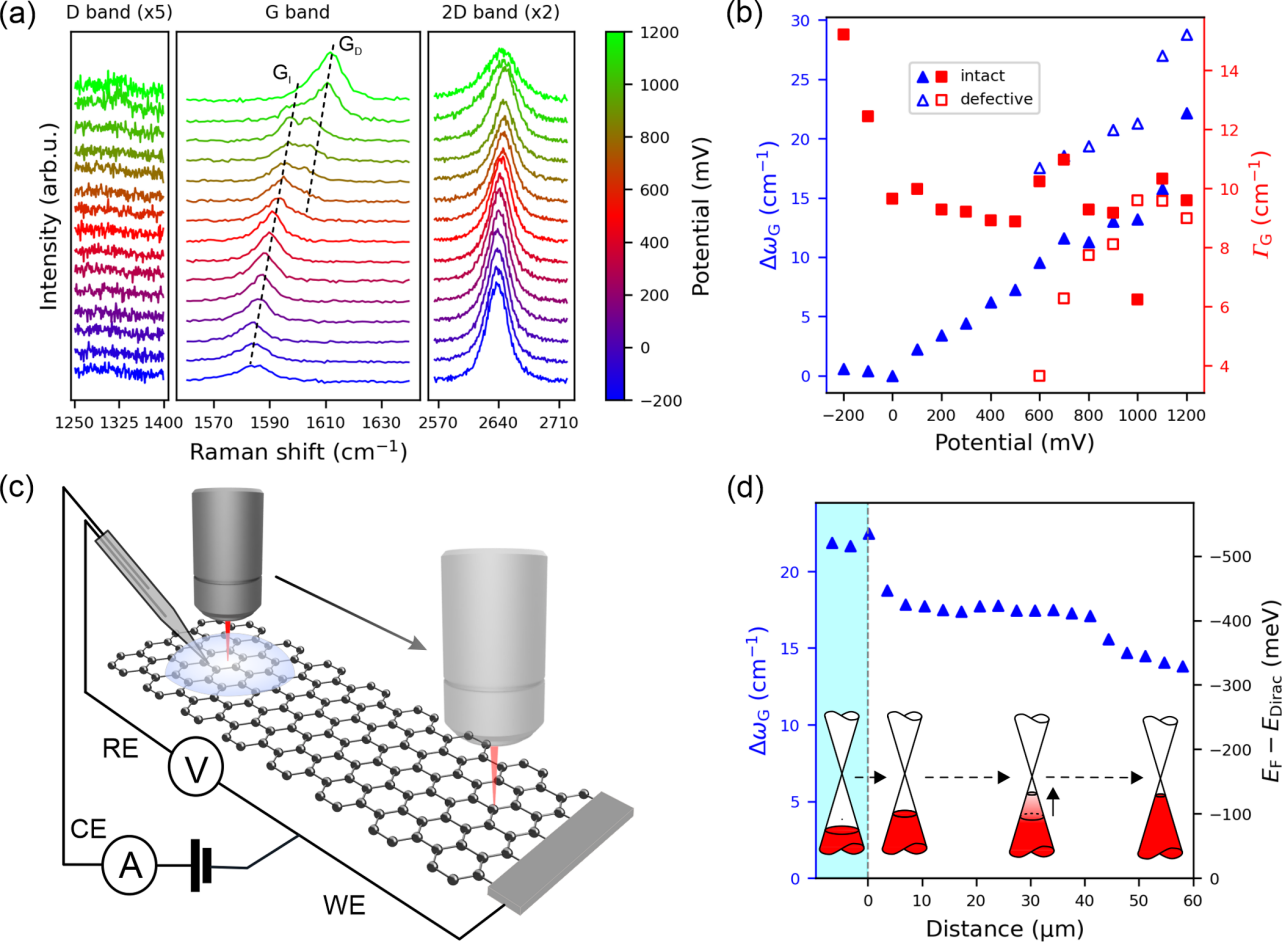
(a) Evolution of the Raman spectra of monolayer graphene with the applied potential (vs Ag/AgCl in 6 M LiCl) inside a microdroplet of 6 M LiCl (aq.) on sample 0. (b) Corresponding *Δω*_G_ (blue triangles) and Γ_G_ (red squares) values in the microdroplet at different applied potentials. Filled/open markers correspond to the intact basal plane/defective regions, respectively. (c) Schematic of *in situ* Raman spectroscopy with localized electrolyte gating, using the working (WE), reference (RE), and counter (CE) electrodes. (d) Spatial evolution of *Δω*_G_ (left axis) and *E*_F_ (right axis) along a line profile from the microdroplet biased to +1200 mV (light blue) to the unbiased graphene on sample 1.

The second-order 2D band is less sensitive to doping than the G band.^8^ As shown in Figure 1a and Supporting Figure S3, it initially upshifts with increasing hole doping in the potential range of –200 mV to +900 mV, followed by a downshift at higher potentials. The hole injection also broadens the 2D band and reduces its intensity due to enhanced hole–hole scattering, which shortens the phonon lifetime and disrupts the coherence of the double-resonance Raman process.^13,32^

To investigate the spatial evolution of *E*_F_ outside the biased region, a constant potential of +1200 mV was applied, and Raman spectra were collected at regular intervals along a line profile starting inside the microdroplet and extending up to 30 to 60 μm onto the unbiased graphene (Figure 1c–d). *E*_F_ is calculated using the formula *Δω*_G_ = |*E*_F_| × 42 cm^–1^ eV^–1^, which reflects the strength of electron–phonon coupling in graphene.^11^ The +1200 mV potential produces reasonably uniform ω_G_ upshift of 22 ± 0.3 cm^–1^ within the biased microdroplet (three measurements), corresponding to *E*_F_ = –530 meV and confirming spatially homogeneous charge injection. Nevertheless, *E*_F_ is most likely underestimated, since the above formula does not discriminate between charge contributions from the intact and defective graphene.^31^

A sharp change in ω_G_ (−3 cm^–1^) and *E*_F_ (+80 meV) is observed upon crossing the microdroplet boundary. Beyond this point, the carrier density gradually equilibrates over distances up to 60 μm, but does not fully return to the original charge state. This incomplete equilibration likely arises from the gradual weakening of the electric field, as graphene’s low density of states provides inefficient electrostatic screening. Only one of the seven independent line-profile measurements performed on different graphene samples (Supporting Figure S4) fully recovers to its original charge state. The average equilibration rate varies from –0.1 to –0.3 cm^–1^/μm (*Δω*_G_) and +2 to +7 meV/μm (*E*_F_). Exponential fits of *Δω*_G_ with distance reveal an average length constant of 3.8 ± 3.2 μm (Supporting Figure S5). The equilibration process is most likely governed by the spatial variations in charge density, arising from nonuniform substrate–induced interactions^5,33,34^ and intrinsic inhomogeneities in graphene.^35,36^

The potential dependence of the spatial dynamics is captured in the line profiles of the G- and 2D-band frequencies and FWHMs at three applied voltages shown in Figure 2. Inside the microdroplet, ω_G_ upshifts by 10, 16, and 24 cm^–1^ from CNP at +600, + 900, and +1200 mV (Figure 2a). At +600 and +900 mV, ω_G_ remains constant, indicating uniform doping, whereas at +1200 mV, variations emerge due to the onset of spatially heterogeneous (photo)­electrochemical oxidation. At the microdroplet boundary, ω_G_ drops abruptly by –5 to –8 cm^–1^, followed by gradual equilibration, whose extent depends on the applied potential. The equilibration rate of the remote gating effects is positively correlated with the applied potential. Importantly, the spatial equilibration of ω_G_ is reversible with respect to the measurement direction, and therefore also in time (Supporting Figure S6). Furthermore, we observe no significant differences when measuring toward or away from the electrical contacts (Supporting Figure S7).

**Figure 2:**
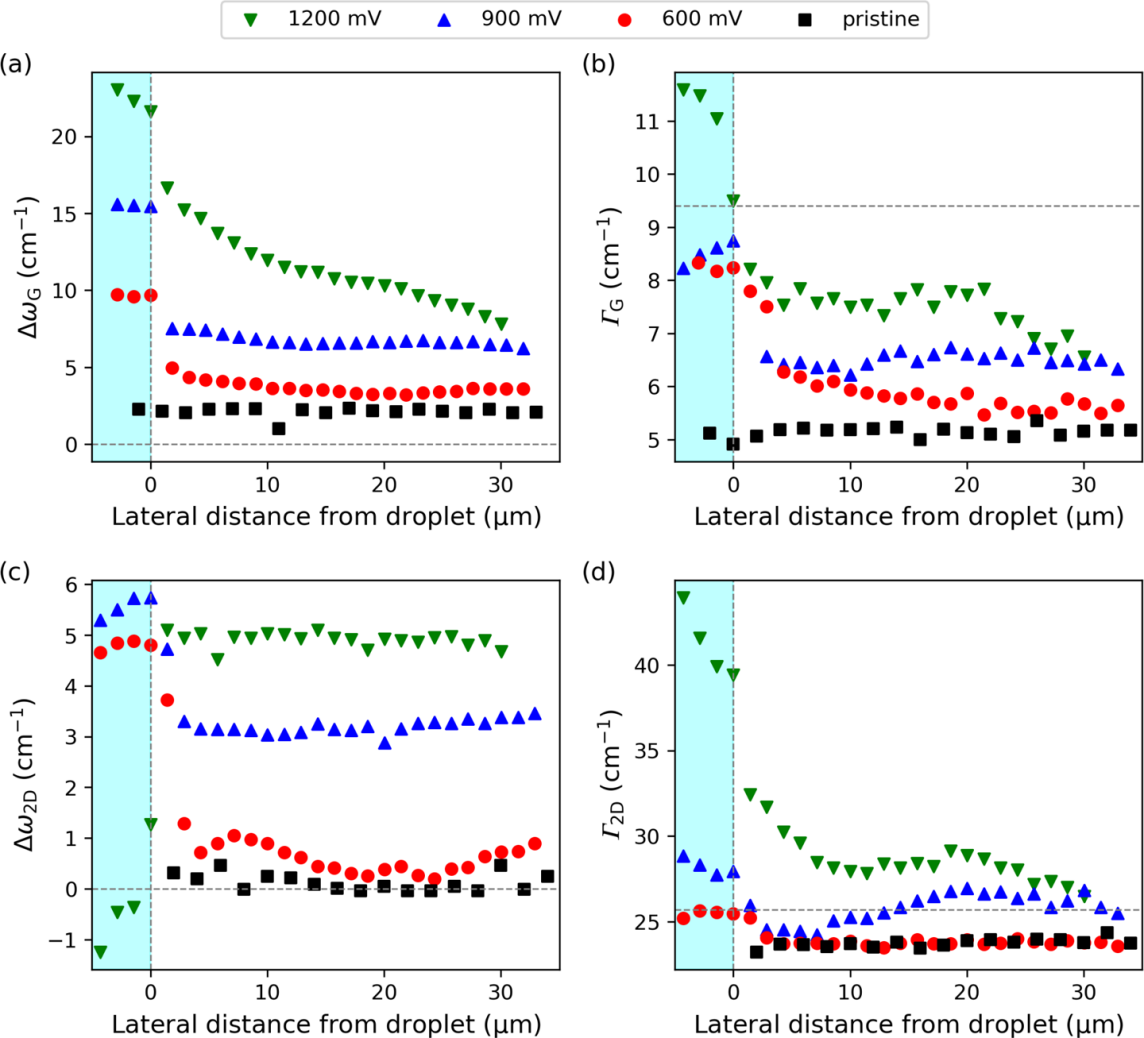
(a) Evolution of (a) *Δω*_G_, (b) Γ_G_, (c) *Δω*_2D_, and (d) Γ_2D_ as a function of distance from the microdroplet biased to +600, +900, and +1200 mV measured on sample 2. Dashed horizontal lines indicate CNP values; dashed vertical lines mark the microdroplet boundary.

Spatial variations in Γ_G_ in (Figure 2b) reflect changes in local carrier density and/or its heterogeneity within the laser spot, assuming uniform strain.^37,38^ Although the carrier density inferred from *Δω*_G_ is higher on biased than on pristine graphene (Figure 2a), Γ_G_ remains elevated proportionally to the carrier concentration, contrary to its expected decrease from its maximum at CNP.^8^ This is because the Γ_G_ of pristine graphene is already close to its reported minimum of ≈5 cm^–1^,^8,12,34,39^ leaving little room for further narrowing by additional carriers. Instead, nanoscale variations in carrier distribution driven by unscreened, substrate-trapped charges broaden the G band. We rule out uniaxial strain as the origin for the Γ_G_ broadening since no discernible changes in the G band are observed at different linear polarization angles of the scattered light within 5 μm from the droplet (Supporting Figure S8).

The 2D band frequency shift from the Dirac point (*Δω*_2D_ = ω_2D_ – ω_2D,Dirac_) amounts to 4–6 cm^–1^ at potentials of +600 and +900 mV within the microdroplet (Figure 2c). In contrast, at +1200 mV, ω_2D_ downshifts due to the enhanced electron–phonon and electron–electron interactions at high doping levels, which give rise to the nonmonotonic behavior of the 2D mode with charge carrier density.^8,9,31^ Upon crossing the microdroplet boundary, *Δω*_2D_ decreases by ≈4 cm^–1^ for +600 and +900 mV, whereas for +1200 mV it flips to higher values. This behavior depends on the laser excitation energy,^40^ as shown in Supporting Figure S9 for the 514 nm laser. The 2D band FWHM (Γ_2D_) increases with the applied voltage inside the droplet (Figure 2d), which is consistent with elevated carrier density.^9^ Spatial equilibration of Γ_2D_ outside the droplet is most pronounced for the highest potential and follows the Γ_G_ evolution in Figure 2b, thus confirming that local charge heterogeneities drive the broadening of the Raman bands in the unbiased region.

2D mode frequency is strongly influenced by substrate interactions,^41^ which are, however, minimal for the SiO_2_ used in the present study. Furthermore, the robustness of our conclusions regarding the equilibration of the Fermi level is chiefly underpinned by the spatial evolution of the G mode frequency, which increases monotonously with carrier concentration, is independent of the laser excitation energy, and does not change upon dielectric screening.^42^

The 2D and G bands of graphene also respond to the mechanical stress,^18,43^ which enables the isolation of doping and strain effects by correlating the 2D and G band frequencies.^44^ Figure 3 shows the ω_2D_–ω_G_ correlation inside the droplet for different applied potentials up to +500 mV (circles), along with the reference trends for pure doping (green dotted lines) and pure strain (red dashed lines). The values initially follow the isostrain line, with a linear slope fit of 0.58 ± 0.05, indicating pure hole doping.^44^ The triangles show the spatial evolution of the modes outside another droplet biased to +1200 mV, which trends toward the potential-dependent data at large distances. The data dispersion inside the droplet (blue triangle) arises from the (photo)­electrochemical oxidation of graphene, but cannot be verified by the D band, whose intensity diminishes at high doping levels.^45^

**Figure 3:**
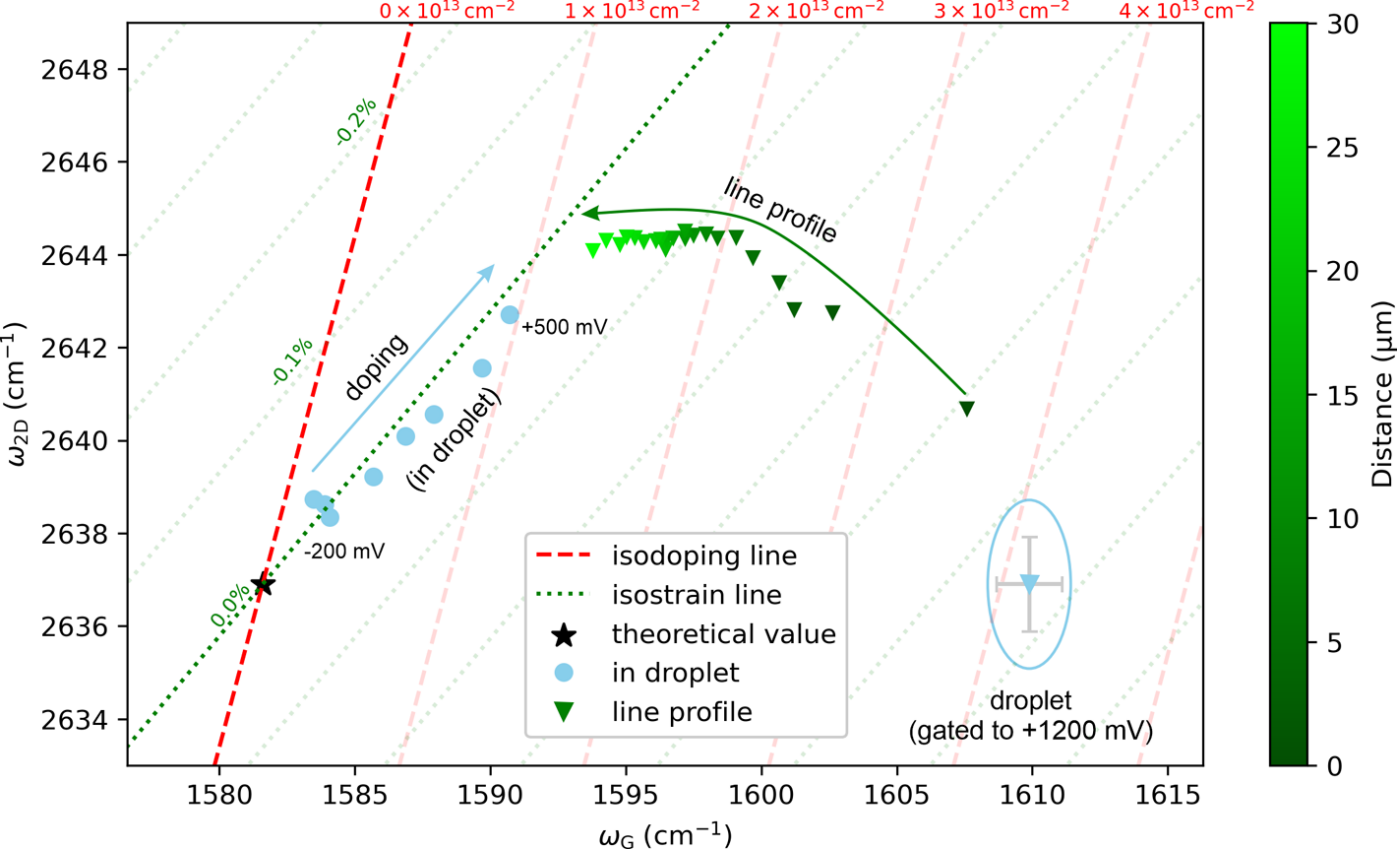
Correlation between ω_2*D*_ and ω_G_. Circles represent measurements inside a microdroplet gated from –200 to +500 mV measured on sample 0. The full data, including the G mode splitting beyond +500 mV, are shown in Supporting Figure S10. Triangles are measurements at different distances from a microdroplet biased to +1200 mV on sample 2; values within the microdroplet are displayed as a mean with standard deviation, enclosed by the blue ellipse. Theoretical isostrain and isodoping trendlines are taken from ref 44.

In summary, *in situ* Raman spectroscopy during localized electrolyte microdroplet gating reveals a gradual spatial equilibration of the Fermi level, which extends tens of micrometers from the biased region, reflecting graphene’s long-range electronic response. However, the Fermi level does not fully return to the original charge state within the examined length scales. These findings reveal that local gating in materials with a low density of states influences electronic properties far beyond the gated region, a crucial consideration for the design and operation of semiconducting and semimetallic devices. This robust experimental platform has broad applicability to other systems in which remote gating effects and spatially dependent carrier transport are central.

## Supplementary Material



## Data Availability

The data and analyses underlying this study are available from the HeyRACK repository at https://doi.org/10.48700/datst.ffkkq-xzg63.

## Notes

Sample preparation, description of the microdroplet spectroelectrochemical setup, data analysis, Raman mapping of graphene before gating, fit of the G band at +600, +700, and +800 mV, evolution of the 2D band with doping, additional line profile data, exponential fitting of the line profile, polarization-dependent G band measurements, G and 2D mode spatial evolution using 514 nm excitation, full 2D–G band correlation. (PDF)
