# Supplementary material for: Spatial Dynamics of the Fermi Level in Electrolyte-Gated Graphene
Source: J Am Chem Soc. 2026 Feb 17;148(8):8054–9. doi: 10.1021/jacs.5c17855 (PMC12964410; doi:10.1021/jacs.5c17855)
Supplement: Supplementary file 1 [file ja5c17855_si_001.pdf]

Supporting Information: Spatial Dynamics of the Fermi Level in Electrolyte-Gated Graphene

Iryna Ivanko,[†] Martin Jindra,[†,‡] Otakar Frank,[†] and Matěj Velický[*,†]

†*J. Heyrovský Institute of Physical Chemistry, Czech Academy of Sciences, Dolejškova 2155/3, 182 23 Prague, Czech Republic*

‡*Department of Physical Chemistry, University of Chemistry and Technology in Prague, Technická 3, 166 28 Prague, Czech Republic*

E-mail: matej.velicky@jh-inst.cas.cz

S1 Methods

S1.1 Sample Preparation

Graphene monolayers were mechanically exfoliated from bulk graphite (NGS Natur Graphit GmbH) onto 90 nm SiO_2/Si wafers (0.001-0.005 Ω·cm resistivity, Siegert) using 1004R-11.0 tape (USI Ultron Systems Inc.). This method yields flakes with low defect density, owing to the absence of stress, heat, and chemical modification, as well as the high quality of the parent crystal.[1–3] The wafers were cleaned by ultrasonication in isopropanol and acetone (3 min each), followed by oxygen plasma treatment for 1 min (40 kHz, 1000 W, 0.9 mbar O_2) in a PICO chamber (Diener electronic GmbH & Co. KG). Graphite exfoliated on tape was pressed onto the cleaned wafers, heated at 100 °C for 2 min on a hotplate, and then peeled off slowly at a low angle. Graphene monolayers were identified by optical contrast relative to the substrate, the 2D/G Raman mode intensity ratio, and the symmetry of the 2D mode.[4,5] Electrical contacts to graphene were made using a silver conductive paint (Electrolube Ltd.) and copper wire (Goodfellow Inc., 99.9% purity).

S1.2 *In situ* Microdroplet Raman Spectroelectrochemistry

Raman spectroscopy was performed using a LabRAM HR spectrometer (Horiba Scientific) with 633 nm laser excitation (0.85 mW power under the microscope) and 514 nm laser excitation (1 mW under the microscope), with a 600 grooves/mm diffraction grating. An Olympus BX-47 confocal microscope with a 100x ultra-long-working-distance objective (NA = 0.7, Mitutoyo) was used to image the electrolyte microdroplet on the graphene surface. The Raman F_{2g} mode of a single-crystal diamond was found to have the full width at half maximum of 3.9 cm^{-1} using this setup, which we consider to be the upper limit for the spectral resolution of the Raman spectra. The spatial resolution of 0.7 µm was determined from a line-scan across a monolayer graphene edge with a 0.3 µm step size, as the Gaussian width of the G band at 50% of its maximum intensity.

Polarization-dependent Raman spectra were acquired using the same configuration, with the

incident-light polarization aligned parallel to the horizontal direction in the field of view and the scattered-light polarization set to 0°, 45°, and 90° relative to the incident-polarization direction. Additional Raman spatial mapping and anti-Stokes/Stokes intensity measurements were carried out on a Witec Alpha 300R spectrometer with 633 nm laser excitation ($<$1.5 mW power).

Electrolyte gating was realized using the Ivium CompactStat.h10800 potentiostat (Ivium Technologies B.V.) in a three-electrode configuration. A Pt wire counter electrode and an Ag wire reference electrode (both Goodfellow Inc., with AgCl subsequently electrochemically deposited on the latter) were housed inside a capillary with a tip diameter of $\approx$1 μm, which was used for pneumatic deposition and stabilization of a 6 M LiCl (Sigma-Aldrich, 99.9%) aqueous electrolyte microdroplet, as described previously.[6] The graphene/microdroplet interface served as the working electrode.

S1.3 Data Analysis

The Raman spectra of graphene were fitted with Lorentzian functions using Python (SciPy) and Mathematica. Data processing and visualization were carried out using OriginPro 2019 (Academic Edition) and Python (Matplotlib, Seaborn).

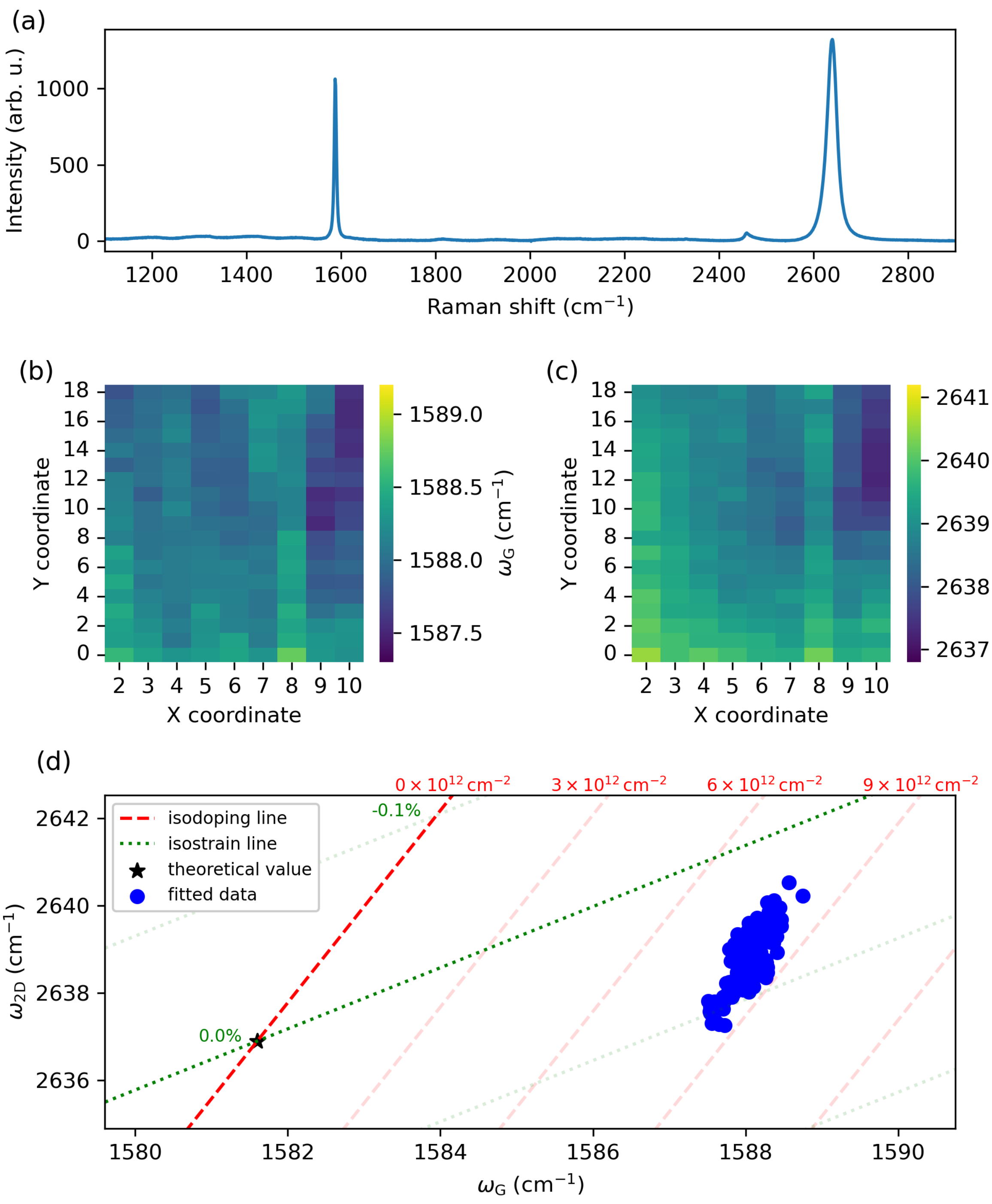

Figure S1: Example of Raman characterization on sample 2 prior to the gating experiments, demonstrating the local spatial homogeneity of the graphene flake. (a) Average spectrum from the Raman map of monolayer graphene. (b–c) Spatial distribution of the G and 2D band frequencies (ω_G and ω_{2D}), respectively. (d) $\omega_{2D} - \omega_G$ correlation analysis revealing small tensile strain ($0.07 \pm 0.01\%$) and uniform doping density ($8.1 \pm 0.3 \times 10^{12}$ cm^{-2}).

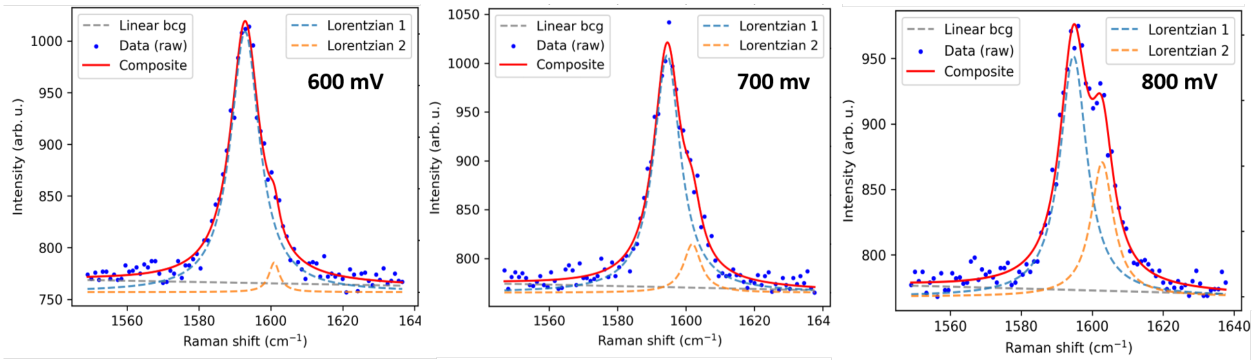

Figure S2: Fitting of the G band of graphene using double-Lorentzian functions. Measured on sample 0.

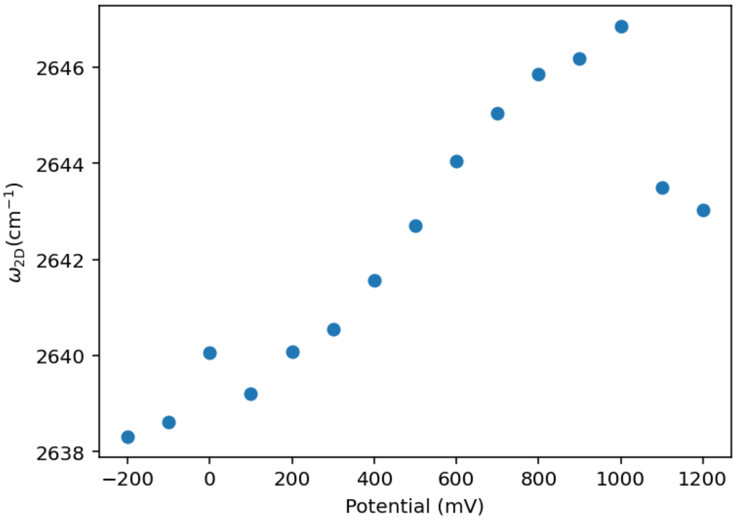

Figure S3: Evolution of ω_{2D} within the microdroplet as a function of the applied potential. Measured on sample 0.

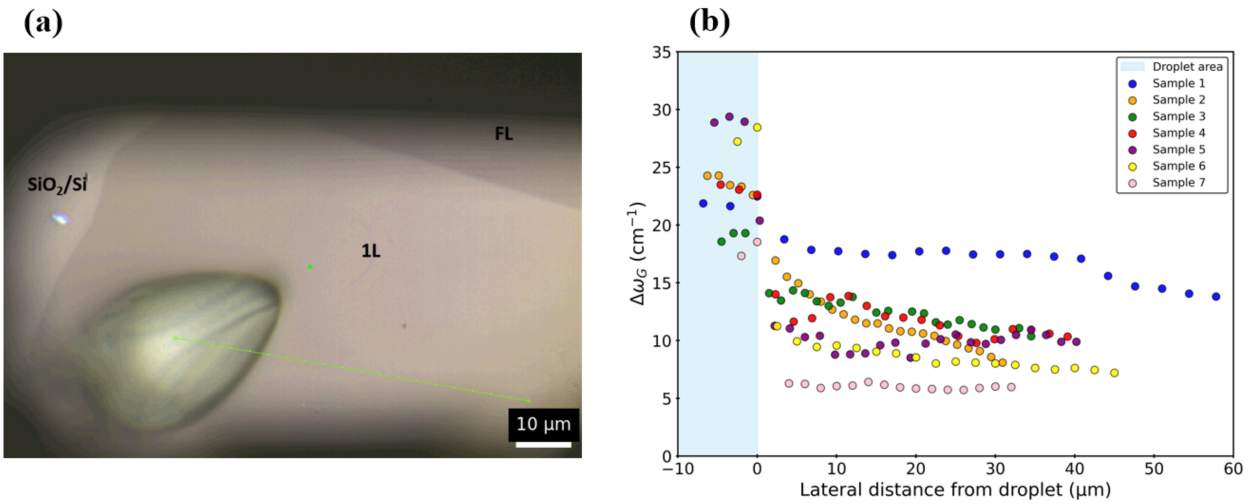

Figure S4: (a) Optical image of monolayer (1L) and few-layer graphene (FL) on an SiO_2/Si substrate with an electrolyte microdroplet (sample 1). (b) Spatial evolution of $\Delta\omega_{\mathrm{G}}$ from the microdroplet biased to +1200 mV onto the adjacent unbiased monolayer graphene on seven different samples. We rule out thermal effects as the driving force for the spatial equilibration, since the local temperature of the substrate, extracted from the anti-Stokes/Stokes intensity ratio of the first-order Raman Si band, increases by $\approx$60 K, which corresponds to a decrease in ω_{G} of less than 1 cm^{-1}.[7] This estimate assumes that the laser induces similar heating effects both in graphene and Si.

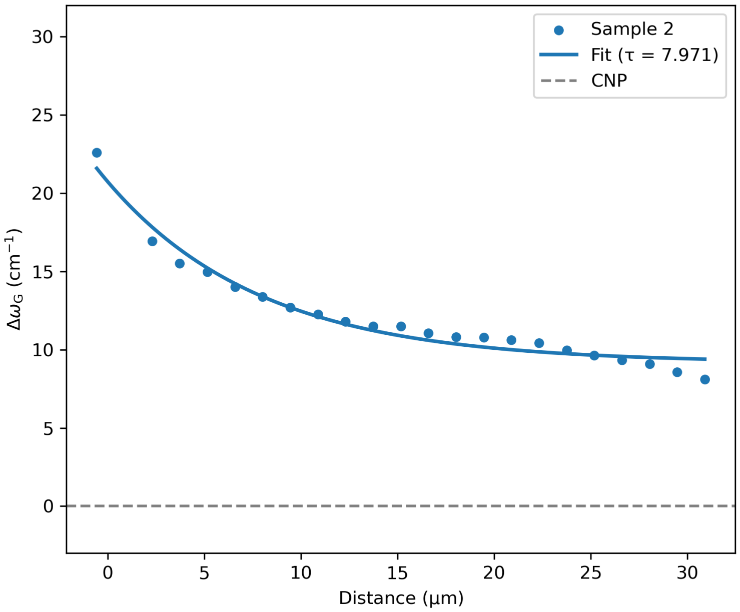

Figure S5: Representative exponential fit of the $\Delta\omega_{\mathrm{G}}$ dependence on distance for sample 2. These fits were used to extract the average length constant at which $\Delta\omega_{\mathrm{G}}$ drops to $1/e$ of the initial value. Although the fits generally provide a good agreement with the data during the initial $\Delta\omega_{\mathrm{G}}$ drop ($\approx$5 μm), they diverge at larger distances for several of the samples, most likely due to the spatial inhomogeneities. Therefore, they serve as convenient phenomenological descriptors of the evolution of equilibration with distance rather than indicators of an underlying physical law.

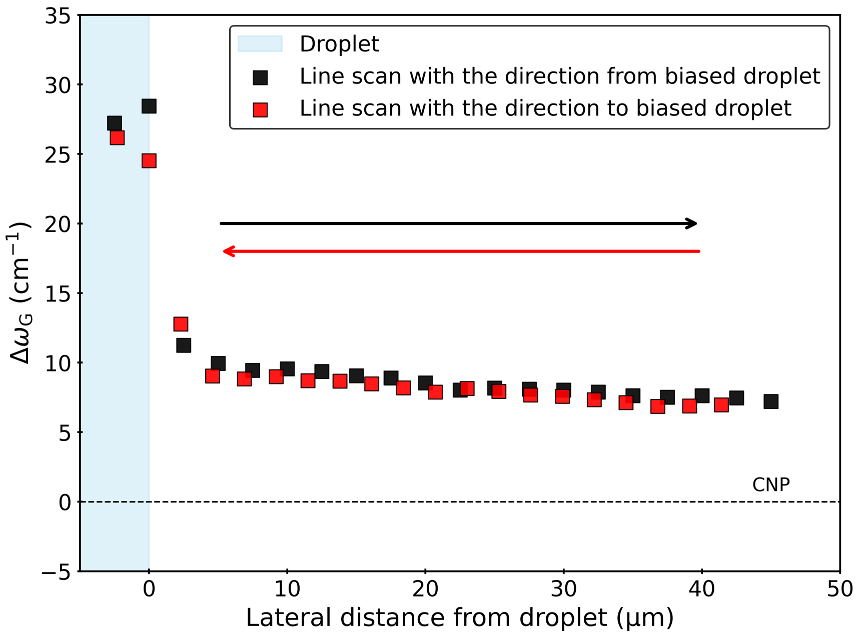

Figure S6: Reversibility of the charge-distribution dynamics, evidenced by successive scans in opposite directions. The black and red data correspond to line profiles measured away from and toward the microdroplet, respectively, biased to +1200 mV. Measured on sample 6.

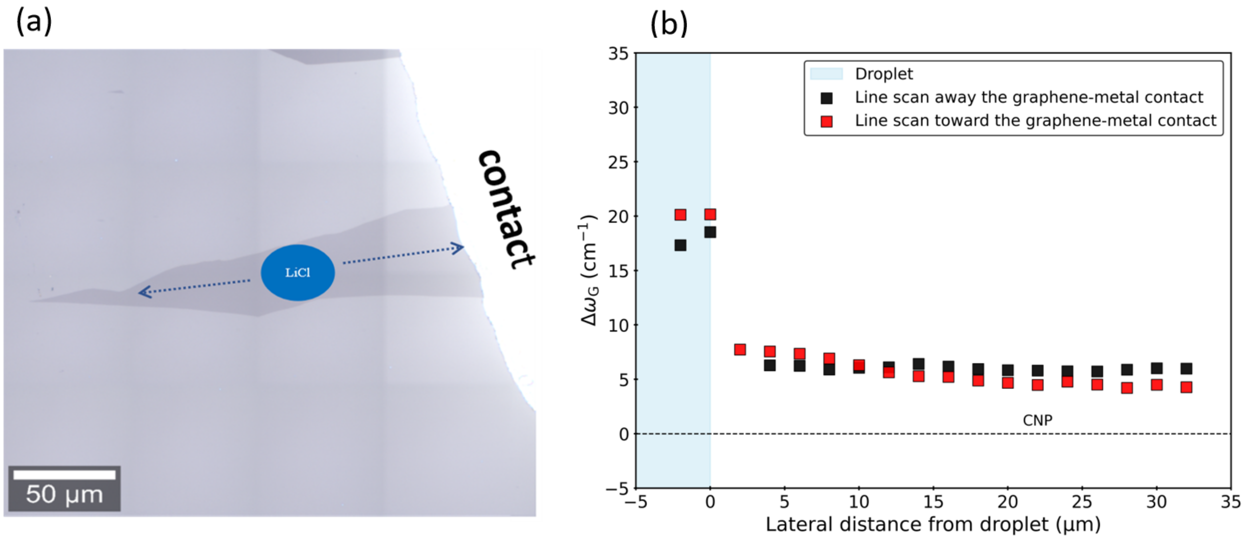

Figure S7: (a) Optical image of a graphene flake, depicting schematically the direction of the line profile with respect to the contact position. (b) Spatial evolution of $\Delta\omega_G$ for a microdroplet biased to +1200 mV, with the black and red data corresponding to measurements taken in the direction away from and toward the electrical contact to graphene, respectively. Measured on sample 7.

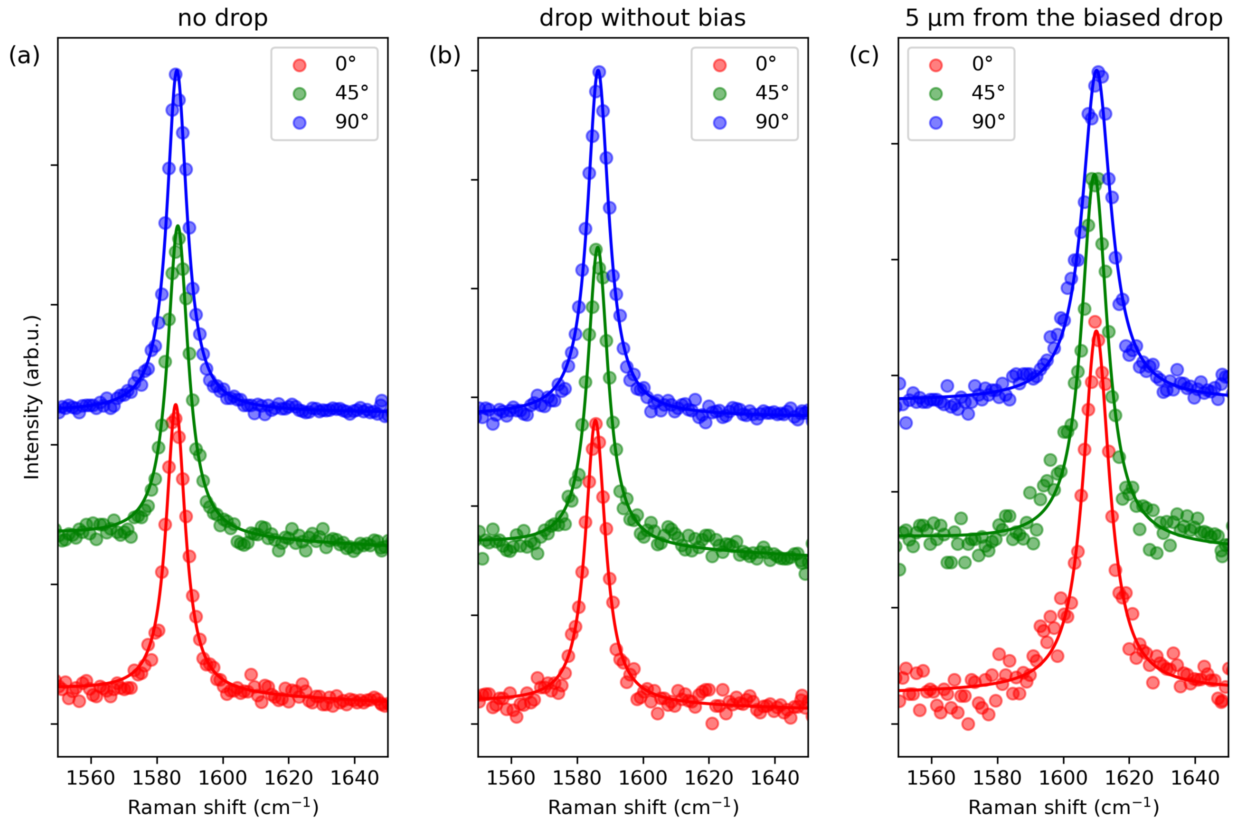

Figure S8: Linearly polarized Raman spectroscopy of monolayer graphene measured at different angles between the fixed incident and variable scattered light (θ = 0°, 45°, and 90°) on sample 8. (a) Spectra recorded prior to microdroplet deposition, (b) Spectra measured at a distance of 5 µm from an unbiased microdroplet deposited on graphene, (c) Spectra measured 5 µm from a microdroplet biased to +1200 mV. Scattered data points represent raw measured data, and solid lines are single Lorentzian fits with a linear background. The absence of polarization angle-dependent changes indicates no G-mode degeneracy lifting associated with anisotropic lattice deformation.[8,9]

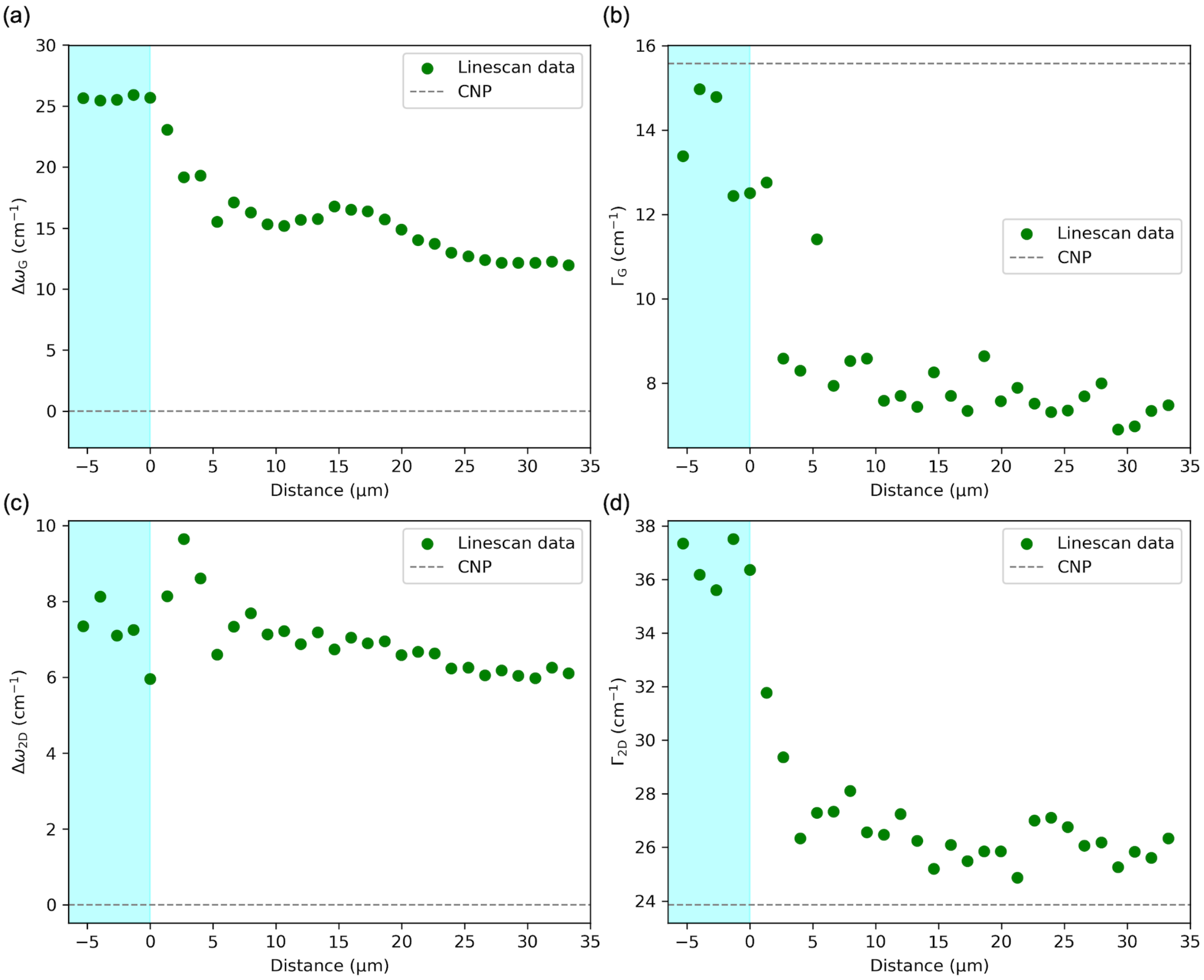

Figure S9: (a) Evolution of (a) $\Delta\omega_G$, (b) $\mathit{\Gamma}_G$, (c) $\Delta\omega_{2D}$, and (d) $\mathit{\Gamma}_{2D}$ as a function of distance from the microdroplet biased to +1200 mV, using the 514 nm excitation wavelength. Dashed horizontal lines indicate CNP values; the vertical blue bands denote the microdroplet region. Unlike for the 633 nm excitation (Fig. 2c in the main text), the decrease in the 2D band frequency within the droplet is not observed here due to the dispersive nature of the 2D band.[10] Measured on sample 9.

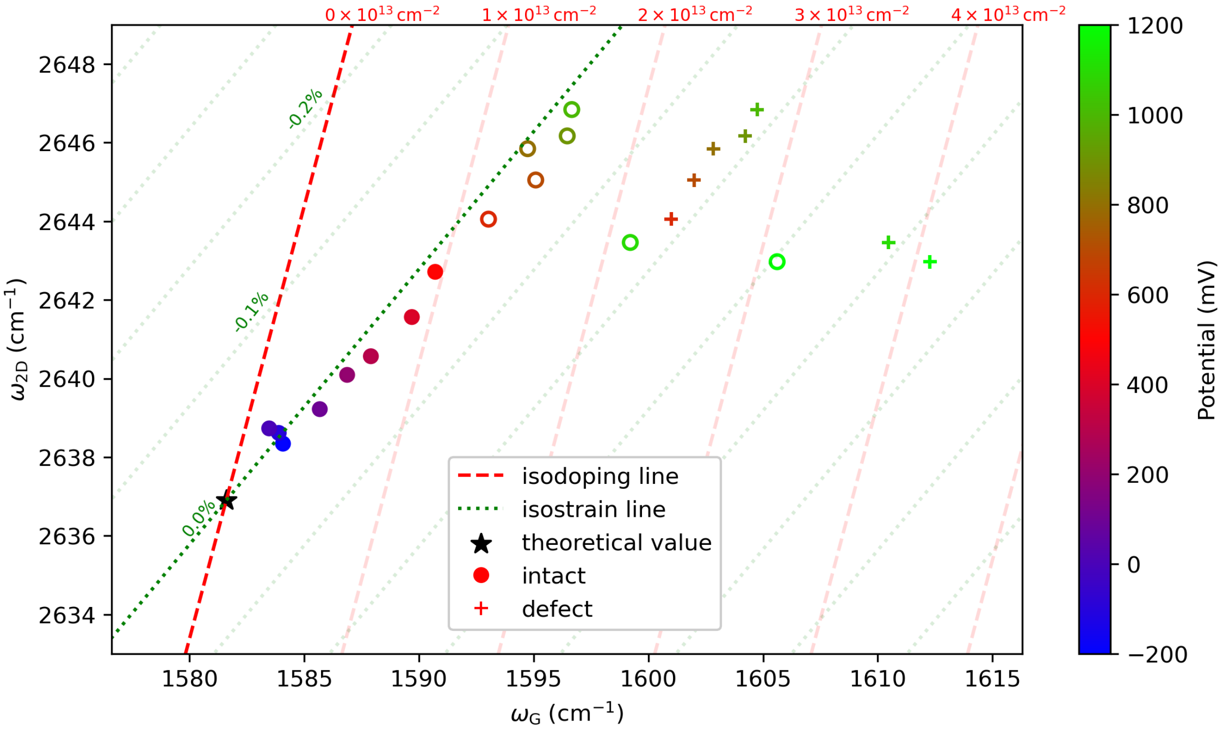

Figure S10: The 2D–G band frequency correlation plot of monolayer graphene doping, showing the full potential range from –200 mV to +1200 mV, measured on sample 0. Starting from +600 mV, the G band splits into the intact G_I (empty circles) and defective G_D (pluses) component.[11] The G_I frequencies continue to track the isostrain direction up to ≈1000 mV, beyond which the G_D component dominates and the ω_G–ω_{2D} correlation becomes unreliable.

References

1. Banhart, F.; Kotakoski, J.; Krasheninnikov, A. V. Structural Defects in Graphene. *ACS Nano* **2011**, *5*, 26–41.

2. Stolyarova, E.; Rim, K. T.; Ryu, S.; Maultzsch, J.; Kim, P.; Brus, L. E.; Heinz, T. F.; Hybertsen, M. S.; Flynn, G. W. High-resolution scanning tunneling microscopy imaging of mesoscopic graphene sheets on an insulating surface. *Proc. Natl. Acad. Sci. U. S. A.* **2007**, *104*, 9209–9212.

3. Ishigami, M.; Chen, J. H.; Cullen, W. G.; Fuhrer, M. S.; Williams, E. D. Atomic Structure of Graphene on SiO_2. *Nano Lett.* **2007**, *7*, 1643–1648.

4. Nemanich, R. J.; Solin, S. A. First- and second-order Raman scattering from finite-size crystals of graphite. *Phys. Rev. B* **1979**, *20*, 392–401.

5. Ferrari, A. C.; Meyer, J. C.; Scardaci, V.; Casiraghi, C.; Lazzeri, M.; Mauri, F.; Piscanec, S.; Jiang, D.; Novoselov, K. S.; Roth, S.; Geim, A. K. Raman Spectrum of Graphene and Graphene Layers. *Phys. Rev. Lett.* **2006**, *97*, 187401.

6. Velický, M.; Bradley, D. F.; Cooper, A. J.; Hill, E. W.; Kinloch, I. A.; Mishchenko, A.; Novoselov, K. S.; Patten, H. V.; Toth, P. S.; Valota, A. T.; Worrall, S. D.; Dryfe, R. A. W. Electron Transfer Kinetics on Mono- and Multilayer Graphene. *ACS Nano* **2014**, *8*, 10089–10100.

7. Calizo, I.; Balandin, A. A.; Bao, W.; Miao, F.; Lau, C. N. Temperature Dependence of the Raman Spectra of Graphene and Graphene Multilayers. *Nano Lett.* **2007**, *7*, 2645–2649.

8. Mohiuddin, T. M. G.; Lombardo, A.; Nair, R. R.; Bonetti, A.; Savini, G.; Jalil, R.; Bonini, N.; Basko, D. M.; Galiotis, C.; Marzari, N.; Novoselov, K. S.; Geim, A. K.; Ferrari, A. C. Uniaxial strain in graphene by Raman spectroscopy: G peak splitting, Grüneisen parameters, and sample orientation. *Phys. Rev. B* **2009**, *79*, 205433.

9. Frank, O.; Tsoukleri, G.; Parthenios, J.; Papagelis, K.; Riaz, I.; Jalil, R.; Novoselov, K.; Galiotis, C. Compression Behavior of Single-Layer Graphenes. *ACS Nano* **2010**, *4*, 3131–3138.

10. Bruna, M.; Ott, A. K.; Ijäs, M.; Yoon, D.; Sassi, U.; Ferrari, A. C. Doping Dependence of the Raman Spectrum of Defected Graphene. *ACS Nano* **2014**, *8*, 7432–7441.

11. Jindra, M.; Velický, M.; Bouša, M.; Abbas, G.; Kalbáč, M.; Frank, O. Localized Spectroelectrochemical Identification of Basal Plane and Defect-Related Charge-Transfer Processes in Graphene. *J. Phys. Chem. Lett.* **2022**, *13*, 642–648.
